# Affirmative citation bias in scientific myth debunking: A three-in-one case study

**DOI:** 10.1371/journal.pone.0222213

**Published:** 2019-09-09

**Authors:** Kåre Letrud, Sigbjørn Hernes

**Affiliations:** 1 Inland School of Business and Social Sciences, Inland Norway University of Applied Sciences, Lillehammer, Norway; 2 Lillehammer Campus Library, Inland Norway University of Applied Sciences, Lillehammer, Norway; Max Planck Society, GERMANY

## Abstract

Several uncorroborated, false, or misinterpreted conceptions have for years been widely distributed in academic publications, thus becoming scientific myths. How can such misconceptions persist and proliferate within the inimical environment of academic criticism? Examining 613 articles we demonstrate that the reception of three myth-exposing publications is skewed by an ‘affirmative citation bias’: The vast majority of articles citing the critical article will affirm the idea criticized. 468 affirmed the myth, 105 were neutral, while 40 took a negative stance. Once misconceptions proliferate wide and long enough, criticizing them not only becomes increasingly difficult, efforts may even contribute to the continued spreading of the myths.

## Introduction

Some misconceptions become engrained in academic publishing and debates. Examples include the low risk of addiction from opioids prescribed for chronic pain [1], the ‘Patient Zero’ supposedly responsible for the U.S. AIDS epidemic [2], the Yerkes-Dodson law [3, 4], the endless behavioral loops of the digger sphex [5], the Learning Styles [6, 7], the Learning Pyramid models [8, 9], and the Hawthorne Effect [10]. Despite fundamental flaws, these claims have proliferated in academic publications for decades, some of them for more than a century. Even though the number of positively identified myths appears to be limited, there is reason to suspect that scientific myths such as these are not a marginal phenomenon. Several unwarranted claims have become part of the scientific corpus [11, 12], and these claims could potentially become entrenched as common knowledge in the way exemplified by the above myths. Citation bias [13–15] contribute to the academic cementation of ideas by favoring studies with positive evidence to those with negative evidence.

Active efforts at countering scientific myth proliferation are required, lest they misinform descriptive and normative deliberations, and clutter encyclopedias, review articles, and topic searches in databases. However, once claims such as these become entrenched in academic discourses, efforts at criticizing them are counteracted by an affirmative citation bias:

We theorize that there are three main ways of citing a critical paper (apart from remaining neutral). Consider the following scenario: A paper sets out to challenge a flawed yet widely distributed theory, and makes a well-argued case against it. If, on the one hand, readers accept the critical arguments and reject the theory, their citation will presumably reiterate the critique. On the other hand, those who disagree with the paper will cite it and make their case for why they choose to sustain the theory, engaging it in a debate. While a third group of readers will cite the critical paper as corroborating the theory, an instance of what Greenberg [15] terms ‘citation diversion’, presumably because they have not read it, or failed to understand it [16–21]. If these three groups are of equal size, those that uphold the model criticized will outnumber those who echoes the critique.

Searching for evidence of affirmative citation bias, we perform case studies of the academic reception of three articles critical of the widely cited yet contentious Hawthorne Effect. By consulting papers citing these critical works, we seek to establish whether, and to what degree the accumulated citations are indeed skewed in favor of the Hawthorne Effect, suggesting the existence of an affirmative citation bias. We shall also search for evidence of citation diversion.

### The Hawthorne effect

The idea of a Hawthorne Effect originated from studies on workplace behavior at the Western Electric Company’s Hawthorne Plant during the 1920s and 1930s, and was primarily based on studies of the relay assembly room, and on informal studies about worker response to changes of lighting [22, 23]. Surprisingly, both higher and lower lighting levels supposedly led to increased productivity. In 1941 Roethlisberger described the altered behavior of workers knowing that they are being observed [24], and in 1953 John French introduced the term ‘Hawthorne Effect’, describing an increase in productivity due to social position and social treatment [22]. ‘The Hawthorne Effect’ is now an ambiguous and vague, yet widely used, term, primarily associated with an observer effect: subjects altering their behavior when aware of being observed [25, 26]. The Hawthorne studies has been in the receiving end of extensive, and sometimes harsh, criticism [24, 27–29], as has the Hawthorne Effect [10, 22, 30].

## Method

We based the case studies on articles arguing against the Hawthorne Effect, interpreted as various observer effects. The selection criteria being that they were unequivocally critical of the effect, that their argumentation was substantial, and that they were extensively cited by peer reviewed articles. We limited our study to reviews and articles indexed by Scopus and Web of Science to ensure they had been peer reviewed, and to facilitate retrieval. Consulting references in, and citations of, Jones’ 1992 seminal critique of the Hawthorne Effect, we sought additional critiques. Among those available to us, we found only two articles that met all the above criteria. We retrieved the available articles indexed in Scopus and Web of science citing these three critiques. A handful were available as pre-prints.

Assessing these citations, we categorized the publications as affirming the effect, as neutral (the category included those not taking a stance on the issue, those that were ambiguous, and those that did not address the effect), and as negative. We separately reviewed the articles before comparing notes. Where our assessments differed we sought an agreement, and if no agreement were reached, we classified the article as neutral.

We found that a few authors cited the source correctly as being critical to the Hawthorne Effect, making the reader aware that it is a contentious theory. However, while taking a neutral, or perhaps even negative stance on the issue, they still approached the Hawthorne Effect as if real when discussing their method or results. We categorized these as de facto affirming.

We also sought to discern whether the authors of the affirmative citations cited the critical articles as affirming the Hawthorne Effect. We found that the majority of affirmative citations simply referred to a critical article when discussing how to avoid the Hawthorne Effect, thus implicitly presenting it as affirmative. However, a handful of these affirmative citations cited the article as a source for how they understood the Hawthorne Effect. Although not serving an argumentative role, the affirmative context nevertheless left the reader with the impression that the cited source did affirm the effect. We chose to classify also these as instances of citation diversion.

## Findings

### Case 1: Franke and Kaul 1978

Franke and Kaul perform the first statistical analysis of the Hawthorne Studies data, and draws conclusions ‘different from those heretofore drawn’ [24]. They do not analyze the data for a Hawthorne Effect, but when they address the effect they concur with several earlier critics on the issue:

Other social scientists have been diverted by the Hawthorne effect, described by Roethlisberger (1941:14): "… If a human being is being experimented upon, he is likely to know it. Therefore, his attitudes toward the experiment and toward the experimenters become very important factors in determining his responses to the situation" (cf. also Dickson and Roethlisberger, 1966, and Bishop and Hall, 1971). This concept of influence upon an experiment through the experiment itself was found either erroneous or misleading by Cook and Campbell (1976), Katz and Kahn (1966), Parsons (1974), and Rubeck (1975). Sommer's (1968) conclusion, that the "errors" called placebo or Hawthorne effect need themselves to be evaluated and understood, is most pertinent. [24]

**Fig 1:**
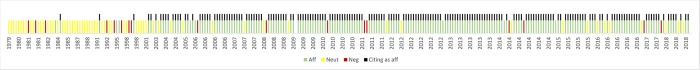
Reception of Franke and Kaul 1978.

Web of Science and Scopus (search date 7 September 2018) indexed in total 285 articles, all published between 1979 and 2018. We were able to retrieve the texts for 277 of these (Table 1; Fig 1).

**Table 1:** Reception of Franke and Kaul 1978.

Citations	1979	1980	1981	1982	1983	1984	1985	1986	1987	1988	1989	1990	1991	1992	1994	1995	1996	1998	1999	2001	2002	2003	2004	2005	2006	2007	2008	2009	2010	2011	2012	2013	2014	2015	2016	2017	2018	Total
Negative			2	1										1	1		1	2							1		1		1	2			2			2		17
Neutral	1	5	7	1	2	1	3	1	4	3	1	2	3		2		1	2	2	2	1	1	1	1	1	1	2			1	1		1	2	3	1	3	63
Affirmative						1	1						1			1	1	1		2		2	6	5	11	13	9	10	14	11	24	18	21	16	14	8	7	197
Citing as affirmative							1						1					1		2		2	6	4	10	13	9	10	14	11	23	18	20	15	14	8	7	189

**Fig 2:**
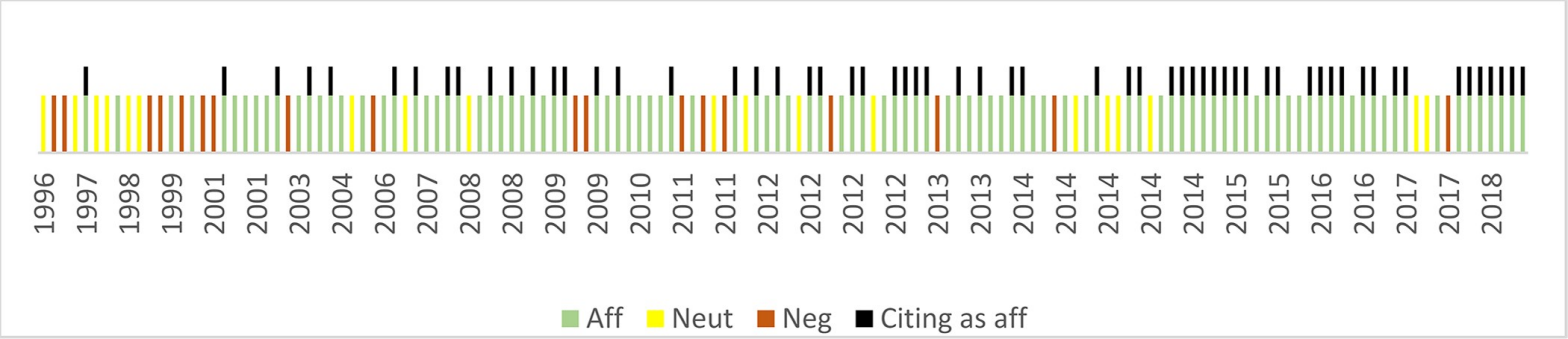
Reception of Jones 1992.

**Fig 3:**
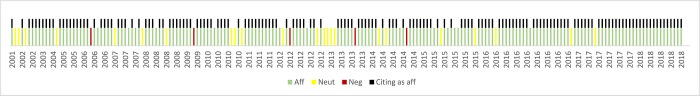
Reception of Wickström and Bendix 2000.

In Figs 1–3, each colored line represents an article. Green lines represent articles affirming the validity of the Hawthorne Effect, while red lines represent those that reject it. Yellow lines are articles that are neutral, ambiguous, or do not address the effect. Black lines mark citation diversion, citing the critical articles as affirming the effect.

17 articles cited Franke and Kaul, while taking a negative stance towards the Hawthorne Effect, and 63 were neutral (of which the majority addressed the analysis of the Hawthorne studies, not the effect). 197 affirmed the Hawthorne Effect, and of these 189 cited Franke and Kaul as affirming the Hawthorne Effect.

### Case 2: Jones 1992

Jones performs an analysis of the data from the relay studies, searching for evidence of the Hawthorne Effect (interpreted as the subjects being aware changes in experimental conditions before or during the experimental period), and finds none. His conclusion:

In this context, I must conclude that there is slender or no evidence of a Hawthorne effect in the Hawthorne Relay Assembly Test Room. Finally, in light of these results, I must also conclude that the Hawthorne effect is largely a construction of subsequent interpreters of the Hawthorne experiments. [22]

Web of Science (search date 24 May 2018) and Scopus (search date 6 September 2018) indexed in total 176 articles citing Jones. We managed to retrieve 141 of these. One contained no citation of Jones 1992, and was rejected, leaving a total of 140 peer reviewed articles, all published between 1996 and 2018. Consulting these 140 articles we found that 19 were neutral on the matter. 18 cited Jones while criticizing the Hawthorne Effect, whereas 103 affirmed its validity. Of the affirmative articles, 60 cited Jones as affirming the Hawthorne Effect (Table 2; Fig 2).

**Table 2:** Reception of Jones 1992.

Citations	1996	1997	1998	1999	2000	2001	2002	2003	2004	2005	2006	2007	2008	2009	2010	2011	2012	2013	2014	2015	2016	2017	2018	Total
Negative	2		1	1	1	2	1				1			2		3	1	1	1			1		18
Neutral	1	2	3							1		1	1			2	2		4			2		19
Affirmative		1	1	1		6	1	3	2		3	4	8	3	5	5	11	9	12	9	9	6	4	103
Citing as affirmative		1				1	1	1	1		1	2	4	3	1	3	7	5	7	7	6	5	4	60

### Case 3: Wickström and Bendix 2000

Citing former reanalyses, Wickström and Bendix [25] argue that the original Hawthorne Studies did not show adequate evidence of the effect. The term, they argue, has come to signify several non-specific outcomes from participating in a study. It is superfluous and truistic, and too vague to be useful:

Instead of referring to the ambiguous and disputable Hawthorne effect when evaluating intervention effectiveness, researchers should introduce specific psychological and social variables that may have affected the outcome under study but were not monitored during the project, along with the possible effect on the observed results. [25]

We were able to retrieve the text of 196 of 198 titles citing Wickström and Bendix published between 2001 and 2018 (search date 9 September 2018). Merely five articles took a critical stance towards the Hawthorne Effect. 23 were neutral, while 168 affirmed the effect. 155 of these 168 articles cited Wickström and Bendix as affirming the Hawthorne Effect (Table 3; Fig 3).

**Table 3:** Reception of Wickström and Bendix 2000.

Citations	2001	2002	2003	2004	2005	2006	2007	2008	2009	2010	2011	2012	2013	2014	2015	2016	2017	2018	Total
Negative						1			1			1	1	1					5
Neutral	2	1			1		2	1		3		2	4	2	2	1	2		23
Affirmative	1	3	3	3	6	7	9	9	6	10	8	11	8	12	16	25	16	15	168
Citing as affirmative	1	3	3	3	6	6	7	8	6	9	8	9	8	11	12	24	16	15	155

## Discussion and conclusion

The ratios between articles that took an affirmative stance towards the Hawthorne Effect and those that rejected it, was for Jones roughly 6:1, and for Franke and Kaul 11:1. The reception of Wickström and Bendix appears to be an outlier at 34:1. The citation diversion group was extensive. Out of 197 affirmative citations of Franke and Kaul, 189 cited the critical articles as affirming the Hawthorne Effect. For Jones, the number was 60 of 103, for Wickström and Bendix 155 of 168.

Considering the numerous citation diversions, a major explanation for the asymmetry between the affirmative articles and the negative articles appears to be not reading, or not understanding, the cited paper. However, we suspect that additional factors may have contributed to the skewed reception:

First, there are incentives for not reiterating the critique if convinced by it. For authors accepting the arguments, the Hawthorne Effect becomes irrelevant, unless, of course, they take issue with the effect specifically. Consequently, we expect them to leave out any mention of the effect, without accounting for their deliberations, nor citing the critical publication, due to common standards of text conciseness and continuity. This can explain the small number of authors reiterating a critical stance towards the theory. Second, the findings may also reflect that the majority of the citers initially were partial to the Hawthorne Effect: Citing while applying the Hawthorne Effect in methodological discussions is presumably more frequent than citing it with the intent of criticizing it.

Of course, to assess whether the three articles were successful at communicating their critique of the Hawthorne Effect, we ought to consider the number of readers that has been dissuaded from believing in and using the Hawthorne Effect in their research. For all we know this group is in majority. It is, however, a silent one. When it comes to academic publishing, the affirming articles are dominant on the issue of the Hawthorne Effect, and are likely the major contributors to the forming of the published consensus. These publications, we surmise, will efficiently recruit new believers in the effect, and in turn new affirmative citations in the literature. The findings not only demonstrate that the three efforts at criticizing the Hawthorne Effect to varying degrees were unsuccessful, but they also suggest that if the intention behind the critiques were to reduce the frequency of affirmations of the claim in the scientific corpus, they may have achieved the very opposite

## Supporting information

S1 File Classification of citing articles. (XLSX)
